# Potential of methacrylated acemannan for exerting antioxidant-, cell proliferation-, and cell migration-inducing activities in vitro

**DOI:** 10.1186/s12906-023-04022-8

**Published:** 2023-06-20

**Authors:** Meng-Han Chou, Yu-Hsu Chen, Ming-Te Cheng, Hung-Chi Chiang, Hou-Wen Chen, Ching-Wei Wang

**Affiliations:** 1 Graduate Institute of Applied Science and Technology, National Taiwan University of Science and Technology, Taipei, Taiwan (ROC); 2 Department of Orthopedic Surgery, Taoyuan General Hospital, Ministry of Health and Welfare, Taoyuan, Taiwan (ROC); 3 Department of Biology and Anatomy, National Defense Medical Center, Taipei, Taiwan (ROC); 4 School of Medicine, National Yang-Ming University, Taipei, Taiwan (ROC); 5 Department of Biomedical Engineering, Chung Yuan Christian University, Taoyuan, Taiwan (ROC); 6 Xinwu Branch, Taoyuan General Hospital, Ministry of Health and Welfare, Taoyuan, Taiwan (ROC); 7 Department of Internal Medicine, Taoyuan General Hospital, Ministry of Health and Welfare, Taoyuan, Taiwan (ROC); 8 Department of Emergency Medicine, Taoyuan General Hospital, Ministry of Health and Welfare, Taoyuan, Taiwan (ROC); 9 Graduate Institute of Biomedical Engineering, National Taiwan University of Science and Technology, Taipei, Taiwan (ROC)

**Keywords:** *Aloe vera*, Acemannan, Antioxidant, Migration, Proliferation, Wound healing

## Abstract

**Background:**

Acemannan is an acetylated polysaccharide of *Aloe vera* extract with antimicrobial, antitumor, antiviral, and antioxidant activities. This study aims to optimize the synthesis of acemannan from methacrylate powder using a simple method and characterize it for potential use as a wound-healing agent.

**Methods:**

Acemannan was purified from methacrylated acemannan and characterized using high-performance liquid chromatography (HPLC), Fourier-transform infrared spectroscopy (FTIR), and ^1^H-nuclear magnetic resonance (NMR). 2,2-diphenyl-1-picrylhydrazyl (DPPH) and 3-(4,5-dimethylthiazol-2-yl)-2,5-diphenyl-2H-tetrazolium bromide (MTT) assays were performed to investigate the antioxidant activity of acemannan and its effects on cell proliferation and oxidative stress damage, respectively. Further, a migration assay was conducted to determine the wound healing properties of acemannan.

**Results:**

We successfully optimized the synthesis of acemannan from methacrylate powder using a simple method. Our results demonstrated that methacrylated acemannan was identified as a polysaccharide with an acetylation degree similar to that in *A. vera*, with the FTIR revealing peaks at 1739.94 cm^−1^ (C = O stretching vibration), 1370 cm^−1^ (deformation of the H-C–OH bonds), and 1370 cm^−1^ (C–O–C asymmetric stretching vibration); ^1^H NMR showed an acetylation degree of 1.202. The DPPH results showed the highest antioxidant activity of acemannan with a 45% radical clearance rate, compared to malvidin, CoQ10, and water. Moreover, 2000 µg/mL acemannan showed the most optimal concentration for inducing cell proliferation, while 5 µg/mL acemannan induced the highest cell migration after 3 h. In addition, MTT assay findings showed that after 24 h, acemannan treatment successfully recovered cell damage due to H_2_O_2_ pre-treatment.

**Conclusion:**

Our study provides a suitable technique for effective acemannan production and presents acemannan as a potential agent for use in accelerating wound healing through its antioxidant properties, as well as cell proliferation- and migration-inducing activities.

## Background

Skin wound healing is a remarkably regulated cellular and molecular mechanism that directly begins after tissue disruption until wound closure [1]. In people with diabetes, wounds need more time to heal. This slow process increases the risk of infections and complications. Patients with diabetes have a 15–25% higher risk of developing diabetic foot ulcers, which severely infect the bone and lead to osteomyelitis [2]. Therefore, proper wound treatment is essential in preventing infection and detrimental complications. Wound dressing provides a suitable environment for wound repair under particular conditions while achieving a complete and cosmetically acceptable appearance [3]. This dressing also protects the wound from the external environment, absorbs wound exudate, and keeps it moist to manage the wound repair process [4]. Recently, biopolymers such as polysaccharides have been increasingly used in wound dressings because of their profusion in the organisms, biocompatibility, and bioactive components [5]. For instance, *Arnebia euchroma* hydrogel [6], extract of milk oil and honey [7], collagen [8], and alginate dressing [9] have been successfully utilized for the local treatment of burns and full-thickness wounds.

*Aloe vera* is a medicinal plant that has traditionally been used to treat a variety of diseases and skin lesions [10]. *A. vera* extract displays antioxidant action, lowering intracellular ROS levels in H_2_O_2_ treated cells and activating antioxidant defense systems and wound healing *via* Nrf2 activation [11]. These activities were found to increase keratinocyte proliferation and migration *in vitro* and improved wound healing [12]. *A. vera* gel not only increases the quantity of collagen production in wounds, but also changes the composition of collagen, promotes collagen cross-linking, thus advancing wound healing [13]. Because 99% of the gel contains water, scientific investigations have shown that it can promote skin elasticity and minimize skin fragility [14]. The polysaccharides in *A. vera* such as acetylated acemannan [15], have been used for various medical problems, such as oral [16], metabolic, and tumor [17] diseases, as well as wound healing [18]. Acemannan is a polysaccharide composed of β-(1,4)-linked highly acetylated mannoses, β-(1,4)-linked glucose, and α-(1,6)-linked galactose [19, 20]. Xing et al. reported that acemannan encourages skin wound healing partly through the activation of the AKT/mTOR-mediated protein translation mechanism, which may represent an alternative therapeutic approach for cutaneous wounds [21]. However, the bioactive components of *A. vera* considerably differ based on their extraction method. Thunyakitpisal et al. reported that the fresh gel extracted from *A. vera* using water extraction and separation (Shodex Sugar KS-804 column) produced 0.04% monosaccharides with the composition Mannose: Glucose: Galactose (65:17:17) [22], whereas extraction from the frozen gel with an ultrafiltration cell membrane (fractionated by ultrafiltration cell with MW cut-off membrane) resulted in 2% monosaccharides with the composition Mannose: Glucose (97:3) [23]. These differences are evident of the need to develop an optimum extraction and/or handling method of *A. vera* to produce maximum acemannan yield. Therefore, this study aimed to develop a suitable method for acemannan production and its characterization for optimum yield and quality, with subsequent potential application in clinical settings, including wound care.

## Methods

### Acemannan preparation

The synthesis of methacrylated acemannan (MACE) was performed using powdered acemannan (ACE; BiAloe®; Mw average = 200 kPa) obtained from Lorand Laboratories (Texas, USA). The powder was dissolved in deionized water (2.5% w/v) at 50 °C and shaken for 8 h until the polymer was fully solubilized. Then, the solution was reacted with 8% methacrylic anhydride (MA; Sigma-Aldrich, Merck, Darmstadt, Germany) and incubated for 6 h at 50 °C. The pH of this reaction mixture was maintained at 8.0 and regularly adjusted with 5.0 M NaOH solution in distilled water. The solution was then dialyzed against distilled water using 12–14 k-CA cut-off dialysis tubing (Thermo Fisher Scientific, Waltham, MA, USA) for 3 d at 25 °C to remove debris and unreacted MA. The solution was then frozen at –80 °C, lyophilized to form a powder, and purified in 85% ethanol for 48 h. The purified acemannan was frozen at –80 °C again and lyophilized at 0.08 mbar and –77.7 °C. The obtained powder was protected from light and stored at 4 °C until further use [24].

### Characterization of acemannan

The acemannan content was measured using high-performance liquid chromatography (HPLC). The powder was dissolved in a 0.5% acidic solution of sodium and shaken for 8 h. The acidic aqueous solution was filtered using a 0.22-μm filter. The acemannan content was detected using an HPLC (SIL10-AS, Shimadzu, Germany) system composed of a TosoHaas column (TKSgel G5000PWxl; TOSOH; Bioscience, LLC), equipped with a SIL-10A injector and UV lamp (Perkin Elmer 235C). Standard acemannan (0.2%) was used as the reference.

Moreover, Fourier-transform infrared spectroscopy (FTIR) was performed to verify the chemical characteristic of acemannan. The samples were analyzed in a Nicolet iS50 FTIR spectrometer (Thermo Fisher Scientific, Waltham, MA, USA) using the attenuated total reflectance technique to obtain 32 scans with a resolution of 2 cm^−1^ at 25 °C.

In addition, ^1^H-Nuclear Magnetic Resonance (^1^H-NMR) spectroscopy was performed using VNMR-J 2.2 c on a Varian VNMRS-500 MHz spectrometer with a 1H-19F/15N-31P 5 mm PFG AutoX DB probe: relaxation delay, 10 s; number of scans, 32; sweep width, 16 ppm; point count, 32 768; pulse angle, 45°; and acquisition time, 2.049 s. All samples were loaded using an automated sample delivery system. For quantitative analysis, the spectra were processed with LB = 0.7 Hz, phase and baseline correction, and weight and Fourier transform function by ACD/Labs 10.0 software; the resonance signals were integrated manually.

### 2,2-diphenyl-1-picrylhydrazyl (DPPH) assay

To prepare a stock solution, 19.7 mg of 2,2-diphenyl-1-picrylhydrazyl (DPPH; #D9132, Sigma, St. Louis, MO, USA) was dissolved in 100 mL methanol and diluted at 1:4.55 to a working solution. The absorbance was measured at ~1.38, λ = 515 nm. Acemannan, malvidin, and CoQ10 were prepared at 45 µM, 54 µM, and 45 µM, respectively. H_2_O was used as a control. Furthermore, 100 μL of each sample was mixed with 900 μL DPPH working solution, stored for 30 min to 24 h at 25 °C in the dark, and centrifuged (for 1 min at 15,000 × *g*), and the absorbance was measured at λ = 570 nm using a microplate reader (Bio-Tek ELX800; BioTek,Winooski, VT, USA).

### Cell culture

Normal mucus-producing epithelial cells of rat gastric mucosa (RGM1) were purchased from Bioresource Collection and Research Center, Taiwan. The cells were cultured in Dulbecco's Modified Eagle Medium: Nutrient Mixture F-12 (DMEM/F12; Sigma-Aldrich; Merck, Darmstadt, Germany) (1:1, vol: vol) and supplemented with 5% heat-inactivated fetal bovine serum (FBS; Hyclone, Logan, UT, USA). The cells were inoculated onto collagen-type I-coated plastic culture dishes (60-mm diameter) at a density of 5 × 10^6^ cells per dish. Approximately 48 h after inoculation, cultured RGM1 cells formed a confluent monolayer sheet.

Cell culture of human fibroblast cells (CG1639), provided by the Bioresource Collection and Research Center, Taiwan, was performed in a T-75 flask (Corning 430,720) with high-glucose DMEM (Gibco, USA) containing 15% FBS (Gibco, USA) and 1% antibiotic-antimycotic (Gibco, USA) in an incubator at 37 °C and a 5% CO_2_ atmosphere. The medium was renewed every 2–3 d.

NCTC clone 929 (L cell, L-929, derivative of Strain L) were purchased from Bioresource Collection and Research Center, Taiwan. L929 cells were grown for three passages from cryogenic storage before seeding for secretion collection. The cells were seeded in 50 mL of high glucose DMEM containing 10% (vol/vol) FBS, 1 mM L-glutamine, 100 U/mL penicillin, and 100 μg/mL streptomycin at a density of ∼6,500 cells per cm^2^ of available surface area. The medium was carefully removed after 7 d of culture and replaced with 50 mL of fresh DMEM medium for the subsequent 7 d.

Furthermore, 3T3-L1 fibroblast cell lines from *Mus musculus* were obtained from Bioresource Collection and Research Center, Taiwan. The cells were grown in DMEM containing 4.5 g/L glucose, 10% (w/v) FBS, and 100 μg/mL penicillin and 0.1 mg/mL streptomycin (complete culture medium) at 37 °C in an incubator with a 5% CO_2_ atmosphere until confluence and ready for further usage.

### Cell proliferation assay

Cell proliferation was analyzed using 3-(4,5-dimethylthiazol-2-yl)-2,5-diphenyl-2H-tetrazolium bromide (MTT; Sigma, St. Louis, MO, USA) powder dissolved to a final concentration of 5 mg/mL in 1 X phosphate-buffered saline (PBS) (137 mM NaCl, 2.68 mM KCl, 10 mM Na_2_HPO_4_, and 1.76 mM KH_2_PO_4_, at pH 7.4). First, the cultured RGM1, L929, and CG1639 cells at a density of 1×10^4^ cells/well were treated with different concentrations of acemannan (0 μg/mL, 39 μg/mL, 1250 μg/mL, and 2000 μg/mL) and incubated at 37 °C in an incubator humidified with 5% CO_2_ for 24 h. The medium from each cell group was then removed and replaced with MTT solution (10 μL per 100 μL medium) and incubated at 37 °C for 4 h. The supernatant was carefully removed and 100 mL dimethyl sulfoxide (DMSO) solution was added to lyse the cells for 10 min. The absorbance was detected using a microplate reader (Bio-Tek ELX-800; BioTek, Winooski, VT, USA) at 570 nm and performed as three independent experiments.

### MTT-based oxidative stress damage assay

We examined the antioxidant effect of acemannan against oxidative stress induced by hydrogen peroxide (H_2_O_2_) in L929 cells. First, the L929 cells were seeded on 96-well culture plates (Corning Costar, NY, USA) and cultured overnight at 37 °C in an incubator humidified with 5% CO_2_ until they reached a density of 1×10^5^ cells/well. Subsequently, 0.8 mM H_2_O_2_ was added to the cultures and incubated for 30 min at 37 °C, followed by 9 µM of acemannan with incubation for another 30 min. After the treatment, MTT solution was added to each well, and the plates were incubated at 37 °C in an incubator humidified with 5% CO_2_ for 1 h. The supernatant was removed, and the cells were lysed completely in 100 mL DMSO. The absorbance was detected using a microplate reader (Bio-Tek ELX-800; BioTek, Winooski, VT, USA) at 570 nm and performed as three independent experiments.

### Cell migration assay

Culture-inserts (ibidi GmbH, Gräfelfing, Germany) plated on 24-well plates (Corning Costar, NY, USA) were used to perform the *in vitro* wound healing assay. The 3T3-L1 cells (inside IBIDI insert) were seeded and cultured in 24-well plates with DMEM culture medium and 10% FBS until they attained a density of 1×10^4^ cells/well. The IBIDI culture inserts were removed, and the two cell islands were washed with PBS to remove debris. The culture plate was incubated overnight at 37 °C in an incubator humidified with 5% CO_2_. The cells were treated with DMEM with different concentrations of acemannan (5 μg/mL, 10 μg/mL, and 25 μg/mL), while the positive control was treated with DMEM supplemented with 10% FBS and the negative control with 5% FBS. Images of the scratch area (wound area) at 0 h were taken using a built-in camera in the microscope (40 X magnification), and then the plate was incubated at 37 °C in an incubator humidified with 5% CO_2_ for 3, 6, and 9 h. Alterations in the injured area after different time points (3, 6 and 9 h) were captured. Data were evaluated using ImageJ software version 1.53t 2022 (National Institutes of Health, Bethesda, MD, USA) to calculate the percent wound area.

### Statistical analysis

All data were analyzed using the one-way analysis of variance or the two-tailed paired t-test of IBM® SPSS® Software Version 18.0 (IBMCorp., Armonk, NY, USA). Significant differences between groups were set at *p < 0.05, **p < 0.01, and ***p < 0.001.

## Results and discussion

### Characterization of acemannan

**Fig. 1:**
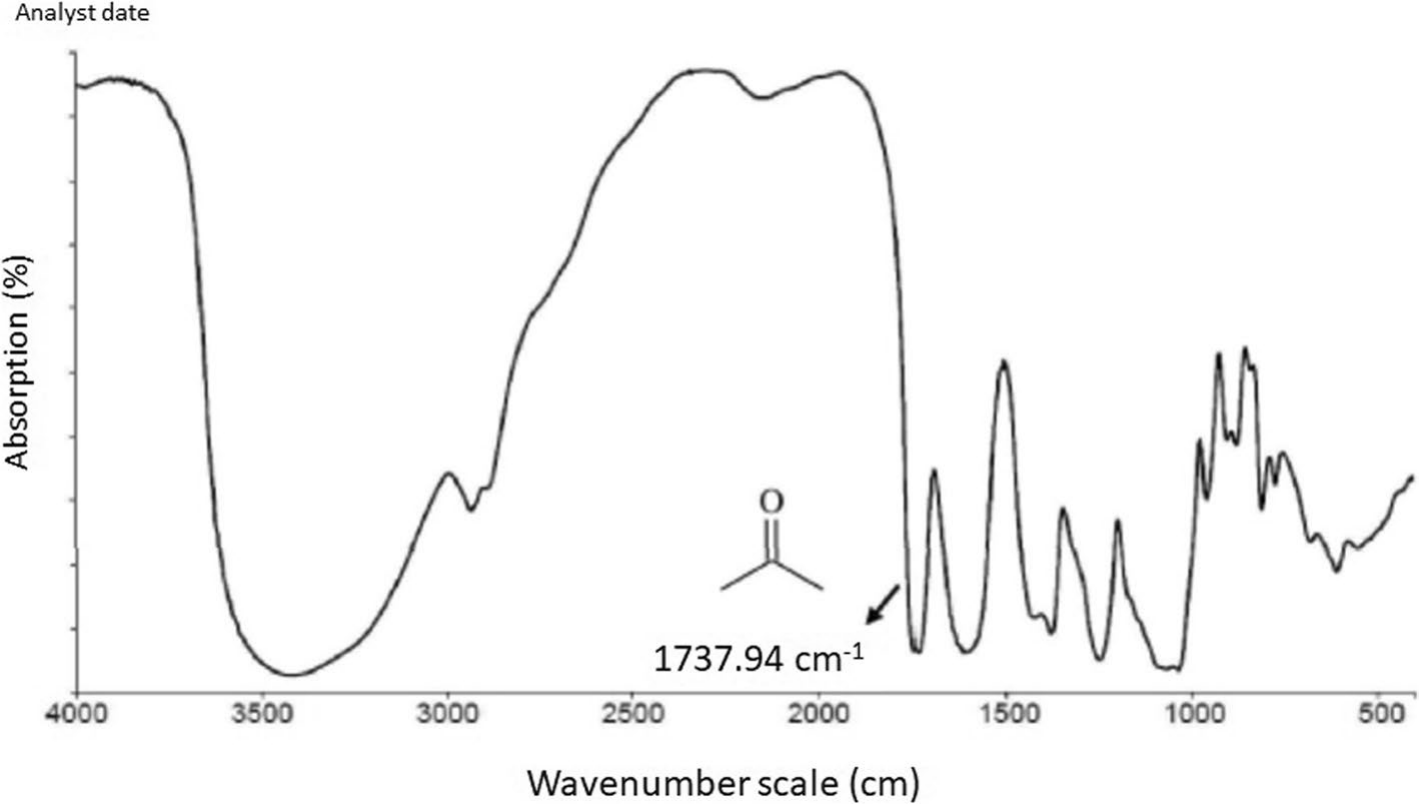
FT-IR analysis spectra of acemannan

The several medical functions of the major polysaccharides in *A. vera* are partially due to acetylated mannan (acemannan). This necessitates characterizing its chemical content to enhance knowledge of its physical, chemical, or biological functions [22, 25–27]. Acemannan is a phytochemical with a long chain polymer of β (1→4) linked galactomannan saccharides [19, 28]. The HPLC analysis data of acemannan from the MACE powder showed similar consistent results with standard acemannan specifications (Table 1); therefore, acemannan in MACE form was used for further experiments. In addition, the acetylation degree of our acemannan (Table 1, O-Acetyl content 526.3 mg/g) was higher than the acceptance criteria, with FT-IR measured peaks at 1739.94 cm^-1^ (C=O stretching vibration), 1370 cm^-1^ (deformation of the H-C-OH bonds), and 1370 cm^-1^ (C–O–C asymmetric stretching vibration) (Fig. 1). These chemical characteristics are used in the identification of acceptable polysaccharides [29].

**Table 1:** Physical characterization of acemannan

Property	Standard acemannan	MACE acemannan	Method
Appearance	Powder	Powder	Materials visual assessment: Consistent with the sample
Color	Off white to pale yellow	Off white	Materials visual assessment: Similar to the sample
Odor	No specific flavor	No specific flavor	Materials visual assessment: Consistent with the sample
Total sugars	≥ 400 mg/g	516.4 mg/g	Acid breaking phenol method
Polysaccharides	≥ 400 mg/g	473.7 mg/g	QB/T2489-2018
O-Acetyl	≥ 500 mg/g	526.3 mg/g	QB/T2489-2018
Acemannan	≥ 60%	66.54%	In-house LC method Q-3–004

**Fig. 2:**
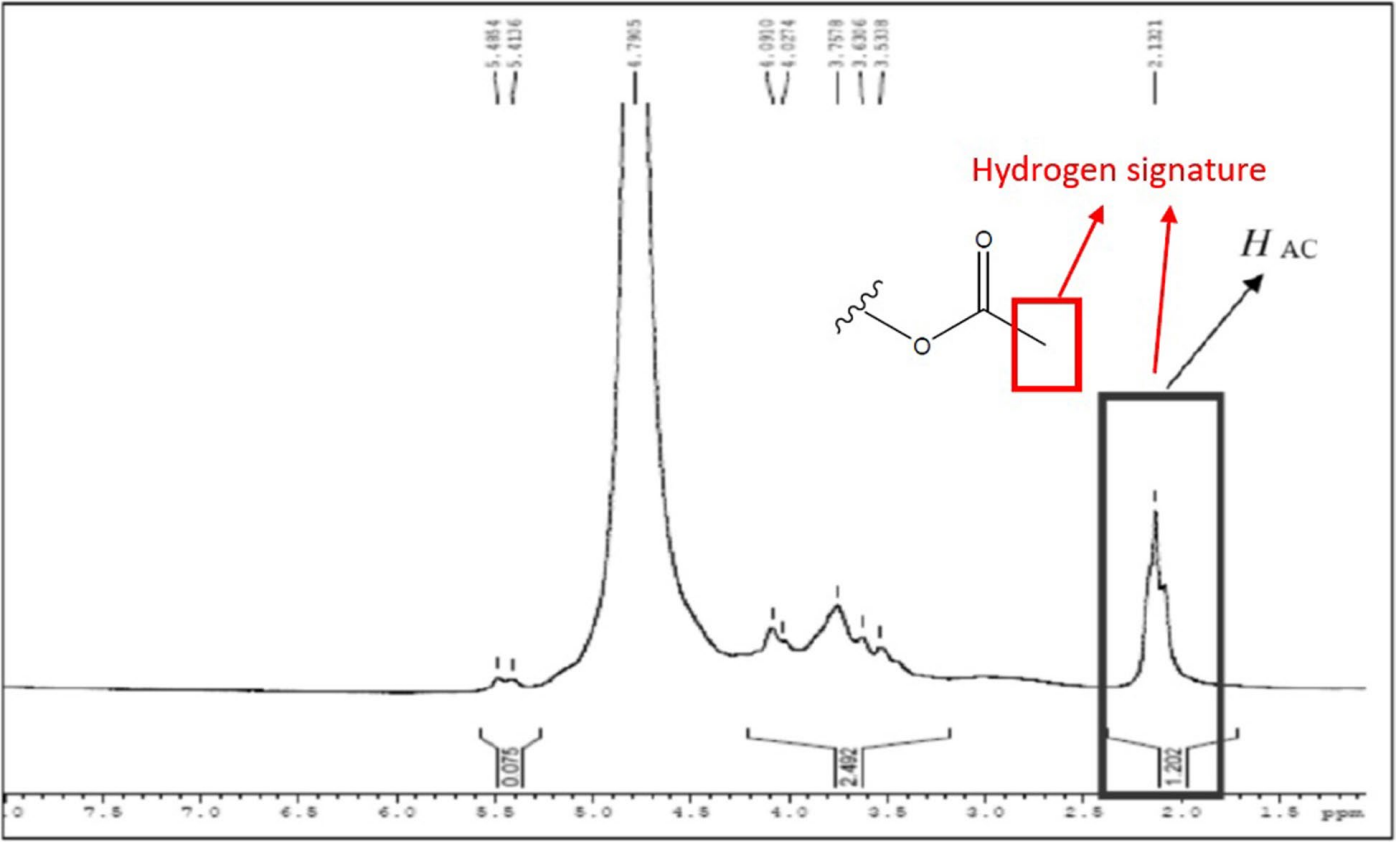
^1^H NMR spectra of acemannan. The red arrows show the functional group detected during the analysis, whereas the black box points to the acetylation degree of acemannan

In addition, ^1^H NMR and IR spectroscopic analyses were performed to identify the acetylation degree of acemannan [19, 30]. Our result showed that the acetyl group proton was found in acemannan and marked as HAC and the acetylation degree of acemannan was 1.202, which is expected in common Aloe species (Fig. 2). In addition, the structure of acemannan in this study was found to be a chain of repeating tetrasaccharide units: -O-(acetyl-D-mannose)-O-(acetyl-D-mannose)-O-(D-glucose)-O-(acetyl-D-mannose) with a single-branched galactose at C6 of the second acetylated mannose residue. The polysaccharide was primarily composed of mannose (65%), glucose (17%), and galactose (17%). In *A. vera*, acemannan has a backbone of β-(1→4)-D-mannosyl residues acetylated at the C-2 and C-3 positions that display a mannose and galactose attached to C-6 [31]. This confirmed that our acemannan consists of mannose (65%), glucose (17%), and galactose (17%). Moreover, the β-(1→4)-glycosidic bond configuration of acemannan is a notable consideration for the therapeutic effects of *A. vera* gel due to the inability of humans to enzymatically degrade these bonds after treatment [32]. The acemannan in *A. vera* is structurally unique, making it a characteristic compound of *Aloe* species among other well-known mannans [19].

### Antioxidant analysis of acemannan

**Fig. 3:**
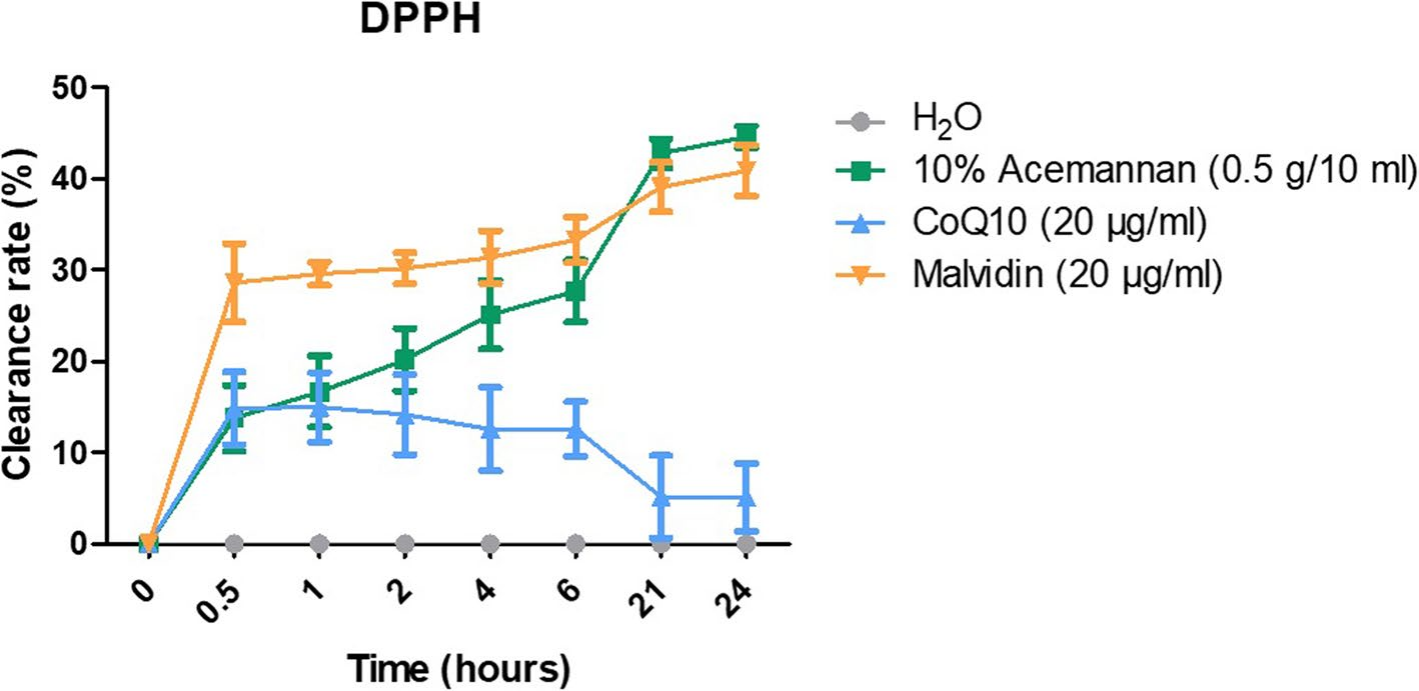
Oxidative radical clearance rates of acemannan, CoQ10, malvidin, and H_2_O

Acemannan possesses antioxidant activity, and this property may be responsible for its therapeutic activities [33]. Our results showed that acemannan had the highest oxidative radical clearance rate of 45% compared with malvidin, CoQ10, and H_2_O (Fig. 3). During the first 30 min, malvidin showed the highest clearance rate, followed by CoQ10 and acemannan. However, in the next hours, CoQ10 showed a decreased trend, but acemannan showed the opposite. At 21–24 h, acemannan showed a higher clearance rate than malvidin. Malvidin is an anthocyanin with antioxidant activity and is commonly found in grapes or blueberries [34, 35], whereas CoQ10 is a vitamin-like substance in the respiratory chain of the mitochondrial membrane that functions as a free radical protector for phospholipids in the intracellular antioxidant system. These two substances are commonly used as antioxidants with potential applications for medical purposes [35, 36]. Based on our results, we confirmed that acemannan is an antioxidant with long-term and stable antioxidant activity and possesses a high clearance rate, representing its highest radical scavenging ability [37]. The acetylation modification of acemannan possibly increases its viscosity and thermal stability due to the stability of the acetyl and hydroxyl groups [38]. Furthermore, the antioxidant potential of acemannan reportedly depends on the concentration of the molecule and the degree of acetylation of the monomeric units [39]. In addition, acetylation may be important for the biological activity, physical properties, and structure of acemannan because the deacetylation of acemannan leads to its ability to induce cell proliferation [40], whereas higher acetylation promotes immunostimulatory activity [41]. Hence, the acetyl group is a prominent part of the structure of acemannan and is responsible for its biological properties, including antioxidant effects [40].

### Cell proliferation assay

**Fig. 4:**
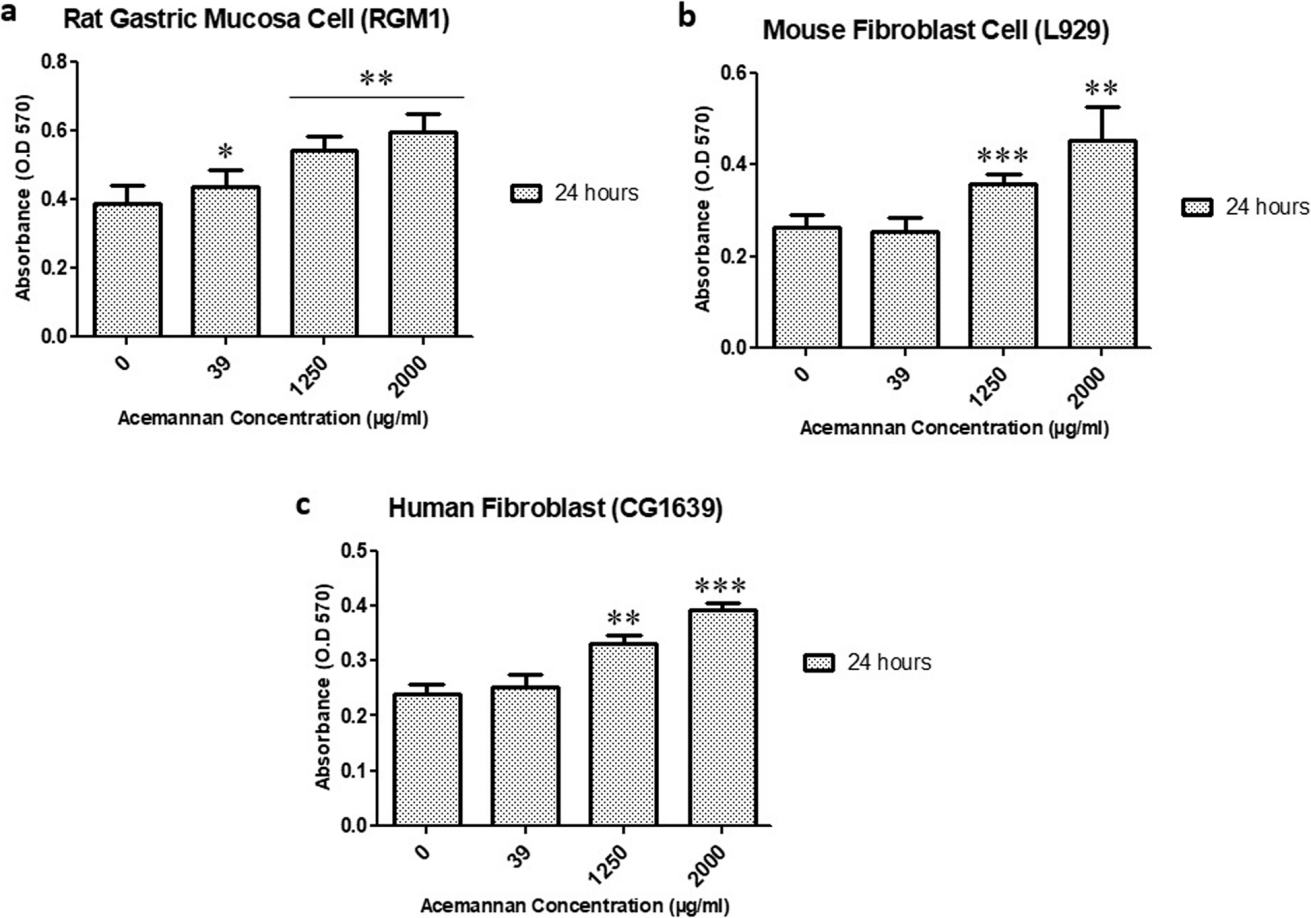
MTT assay for cell proliferation activity after acemannan treatment at different concentrations. **a** Rat gastric mucosa cells (RG); **b** Mouse fibroblast cells (L929); **c** Human fibroblast cells (CG1639). Significant differences between groups were set at *p < 0.05, **p < 0.01, and ***p < 0.001

Wound healing is a complex process in response to injury, which is purposed at reconstructing damaged tissues with significant coordination of connective tissue repair, re-epithelialization, and angiogenesis [30]. To generate new tissue and heal wounds, fibroblasts proliferate to increase cell numbers and produce several extracellular matrix proteins and growth factors [30, 42]. Thus, an alternative component, such as acemannan in *A. vera*, can enhance cell proliferation for wound recovery [18]. Our results showed that after 24 h of treatment, the proliferation activity of RGM1, CG1639, and L929 cells increased with increasing acemannan concentration (Fig. 4). A 2000 µg/mL concentration of acemannan was the optimal concentration for inducing cell proliferation. This suggests that acemannan can induce the proliferation of cells and can be used for similar medical-related purposes.

### Cell-based analysis for antioxidant capacity

**Fig. 5:**
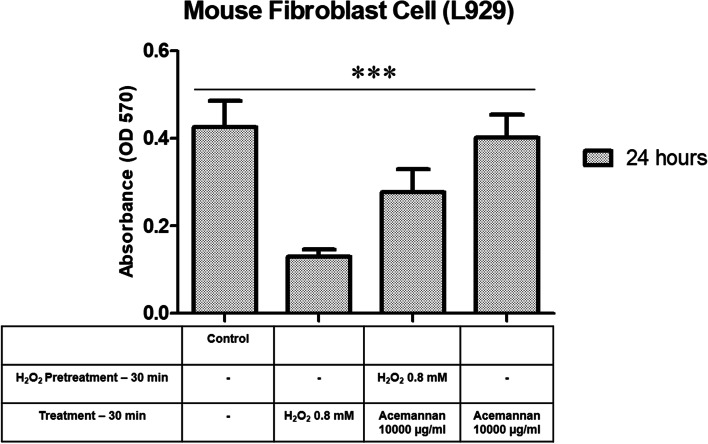
Antioxidant effect of acemannan in L929 cells pretreated with H_2_O_2_. Significant differences between groups were set at *p < 0.05, **p < 0.01, and ***p < 0.001

MTT assay was performed to assess the antioxidant activity of acemannan on cells after induction of cell damage *via* pretreatment with H_2_O_2_. Excessive free radicals may degrade ECM proteins, leading to increased severity of various human diseases such as diabetes mellitus, aging, and chronic wound healing [43]. H_2_O_2_ pretreatment triggers oxidative stress in cells, resulting in cell damage and decreased cell viability [44]. In our experiment, the cell damage induced by H_2_O_2_ was distinguished by a decrease in absorbance compared with the control (Fig. 5). The results demonstrated that the addition of acemannan to the cell culture initiated cell recovery represented by the increase in absorbance. Moreover, acemannan administration only (without H_2_O_2_ pretreatment) showed the highest absorbance compared with acemannan-H_2_O_2_ pretreatment. This suggests that acemannan treatment can increase cell viability in damaged or healthy cells, owing to its antioxidant effect [45].

### Cell migration analysis

**Fig. 6:**
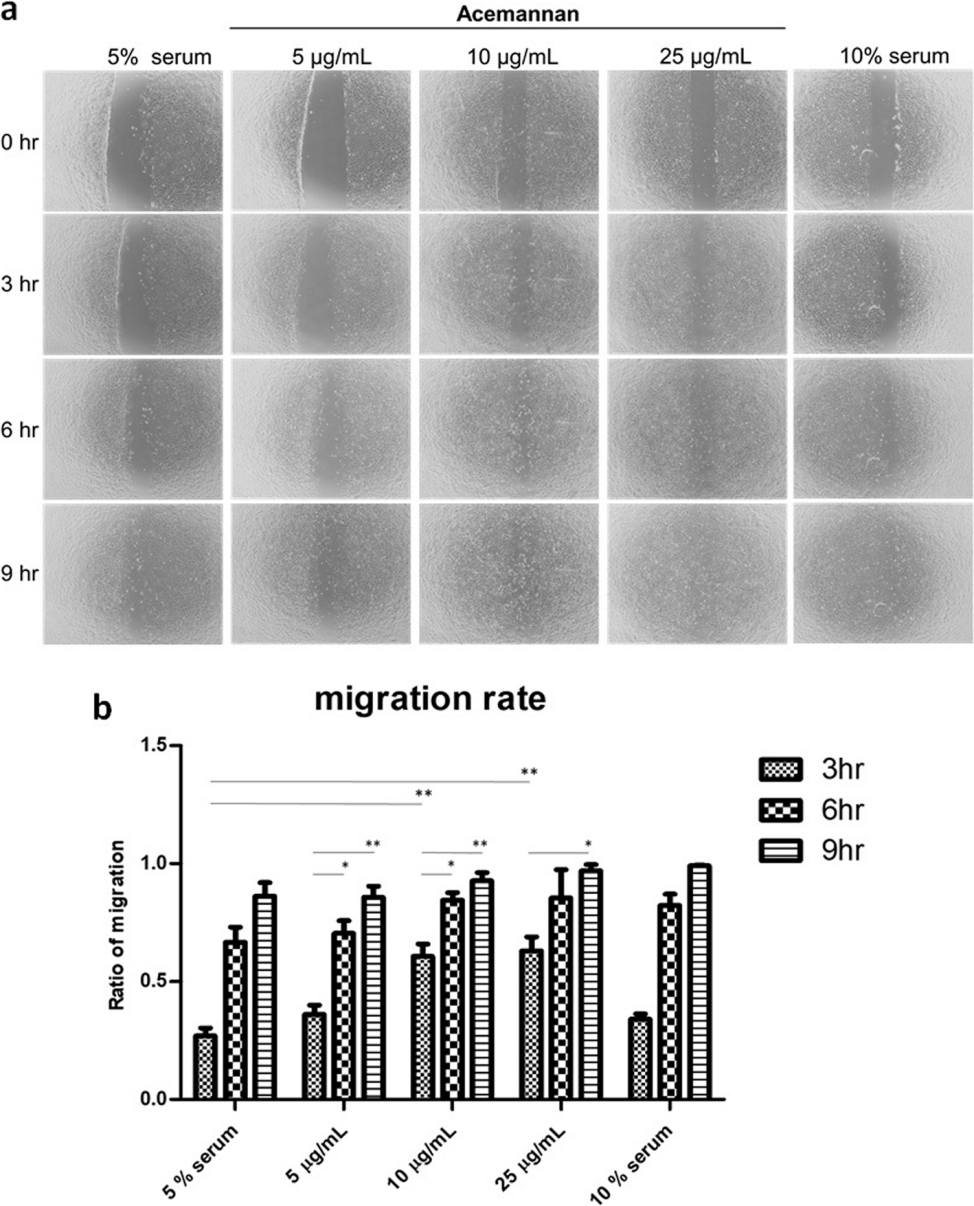
Cell migration rate in 3T3-L1 cell lines with different concentrations of acemannan. **A** The migration of cells from 0 to 9 h following acemannan treatment; **B** The migration cell rate: 25 µg/mL acemannan shows the highest migration rate of the cells compared to other concentrations. Significant differences between groups were set at *p < 0.05, **p < 0.01, and ***p < 0.001

The primary goal of epithelial cells in wound healing is to restore the broken epithelial barrier after injury [46]. Therefore, cell migration is an important process in wound repair. During this stage, the epithelial cells migrate through the wound bed, and cell-cell adhesion is maintained to close the epithelial barrier properly [47]. Our results showed that 25 µg/mL acemannan treatment induced the highest level of cell migration after 3 h, followed by a significant increase at 6 h and 9 h later (Fig. 6A and B). Hence, acemannan is a promising agent for wound healing owing to its cell proliferation- and migration-inducing activities [12].

A similar study was also conducted by Tavakolizadeh et al., which demonstrated strong tissue adhesiveness (adhesive strength of up to 48 N m-1) and outstanding therapeutic qualities (cell viability of A375 cells >80%; remarkable healing of >60% in 14 and >98% in 21 d) of *A. euchroma* hydrogel on wounds [6]. Teplicki et al. [10] used another traditional plant and showed that *A. vera* gel displayed considerable stimulatory effects on fibroblast and keratinocyte cell proliferation and migration, as well as strong protective benefits against preservative-induced keratinocyte death. Those abilities in plants are likely due to numerous element contents, including polyphenols, flavonoids, phenolic acids, and acemannan which are responsible for suppressing free radicals and antioxidant effects in plants [48, 49]. Acemannan from *A. vera* hydrogel specifically can enhance wound healing *in vivo* by promoting cell proliferation and differentiation [50]. This substance also has a variety of biological roles, including wound healing, antimicrobial, antiviral, and anticancer [6, 49–51] properties. Given its properties, acemannan can be used to stimulate cell proliferation, migration, and free radical recovery. It appears that using acemannan as supplemental therapy alongside current treatments can improve wound healing and improve societal health.

## Conclusions

In summary, we successfully optimized the synthesis of acemannan from methacrylate powder using a simple method and adequate characterization techniques. Our results suggest that acemannan is a polysaccharide that has high and stable antioxidant properties with 24-h clearance activity compared to CoQ10, malvidin, and water. Acemannan was also able to stimulate cell proliferation and migration, as well as promote cell recovery after damage caused by H_2_O_2_. We believe that our study provides a better technique for acemannan production, as well as shows the potential of acemannan in accelerating the wound healing process.

## Data Availability

All data generated or analyzed during this study are included in this published article.

## Abbreviations

DPPH: 2,2-Diphenyl-1-picrylhydrazyl
MTT: 3-(4,5-Dimethylthiazol-2-yl)-2,5-diphenyl-2H-tetrazolium bromide
MACE: Methacrylated acemannan
HPLC: High-performance liquid chromatography
FTIR: Fourier-transform infrared spectroscopy
^1^H-NMR: ^1^H-nuclear magnetic resonance
RGM1: Rat gastric mucosa
CoQ10: Coenzyme Q10
